# Physiological controls of large‐scale patterning in planarian regeneration: a molecular and computational perspective on growth and form

**DOI:** 10.1002/reg2.54

**Published:** 2016-04-28

**Authors:** Fallon Durant, Daniel Lobo, Jennifer Hammelman, Michael Levin

**Affiliations:** ^1^ Department of Biology, Allen Discovery Center at Tufts University, Tufts Center for Regenerative and Developmental BiologyTufts UniversityMA02155USA; ^2^ Department of Biological SciencesUniversity of MarylandBaltimore County, 1000 Hilltop CircleBaltimoreMD21250USA

**Keywords:** Anatomy, bioelectricity, computation, gap junctions, ion channels, morphogenesis, morphology, planaria, regeneration

## Abstract

Planaria are complex metazoans that repair damage to their bodies and cease remodeling when a correct anatomy has been achieved. This model system offers a unique opportunity to understand how large‐scale anatomical homeostasis emerges from the activities of individual cells. Much progress has been made on the molecular genetics of stem cell activity in planaria. However, recent data also indicate that the global pattern is regulated by physiological circuits composed of ionic and neurotransmitter signaling. Here, we overview the multi‐scale problem of understanding pattern regulation in planaria, with specific focus on bioelectric signaling via ion channels and gap junctions (electrical synapses), and computational efforts to extract explanatory models from functional and molecular data on regeneration. We present a perspective that interprets results in this fascinating field using concepts from dynamical systems theory and computational neuroscience. Serving as a tractable nexus between genetic, physiological, and computational approaches to pattern regulation, planarian pattern homeostasis harbors many deep insights for regenerative medicine, evolutionary biology, and engineering.

## The problem of form in planaria

### Introducing the flatworm model species

Planaria are free‐living tripoblastic animals whose bodyplan (Martín‐Durán et al. 2010) exhibits three major axes of polarity: dorsal/ventral, anterior/posterior (AP), and medial/lateral (with a cryptic left/right asymmetry) (Nogi et al. 2005). Their complex anatomies include musculature of two distinct types (Kobayashi et al. 1998), digestive system (Newmark & Alvarado 2002), and reproductive system (Hyman 1951) complete with germ cells (Wang et al. 2010). Moreover, they have a true centralized brain (Sarnat 1985; Pagán 2014), which produces a continuous brain wave frequency as observed by electroencephalogram (Aoki et al. 2009), and central nervous system (CNS) (Cebrià 2008). Planaria exhibit complex behaviors, including decision‐making (Inoue et al. 2015) and lateralization (Corning 1964). They also have robust sensory capabilities and organs (Hyman 1951), mediated by photoreceptors (Carpenter et al. 1974) and various receptors in their auricles (MacRae 1967; Asano et al. 1998). Aside from chemical and mechanical cues, they also have the ability to sense electrostatic (Brown 1962), radioactive (Brown & Park 1964), and magnetic aspects (Brown 1962, 1966) of their environment.

The most unique aspect of this model species is that they are true champions of regeneration, able to replace any body part that is amputated (Morgan 1898) while recapitulating proper organ size (Hill & Petersen 2015). They also continuously remodel their bodies, maintaining proportions suitable to available food supply (Oviedo et al. 2003; González‐Estévez & Saló 2010; Forsthoefel et al. 2011; Beane et al. 2013), and appear to have solved the problem of aging by continuous regeneration of their soma. While the implications of this for human regenerative medicine and stem cell biology have been expertly discussed (Sanchez Alvarado 2007; Moraczewski et al. 2008; Rossi et al. 2008; Gentile et al. 2011), we focus here on another aspect highlighted by these remarkable creatures: the control of large‐scale shape.

### Big questions of planarian regeneration: beyond cell differentiation

**Figure 1:**
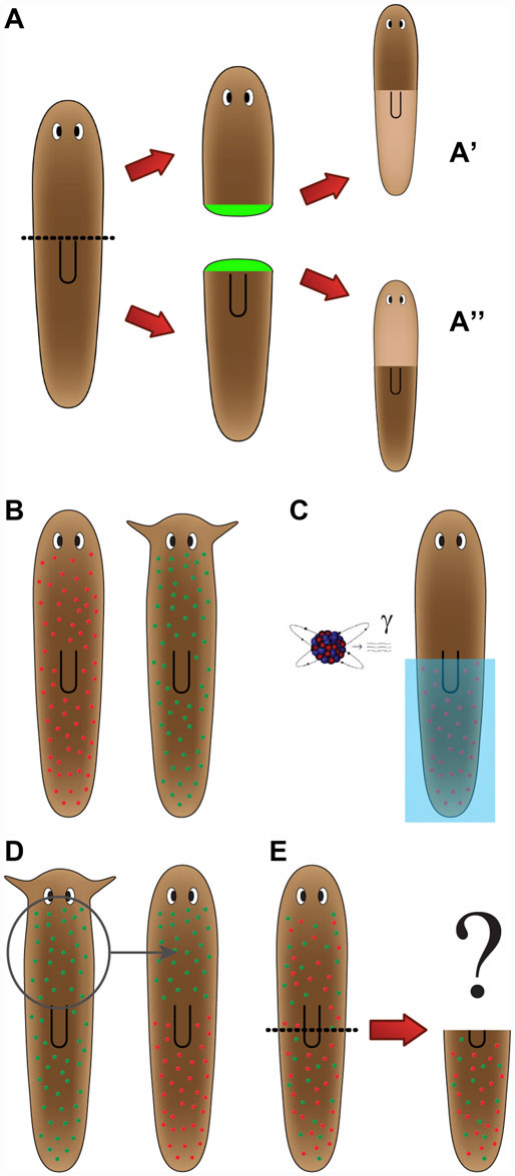
Fundamental questions of large‐scale patterning in planaria. (A) Global decision‐making in planaria. After bisection, the two resulting blastemas must make very different structures: a tail (A′) or a head (A″). However, the two sets of cells were at the same position in the worm and thus start out with the same information prior to the cut. This illustrates the global nature of regeneration, because the blastema needs information from the rest of the fragment to correctly make the AP anatomical fate decision. (B) Mixing neoblasts to probe target morphology. *Schmidtea mediterranea* (left, rounded head) and *Polycelis felina* (right, pointed head) are planarian species with different head morphologies. (C) Half the neoblasts of the rounded‐head worm are killed using irradiation and a lead shield. (D) Half the neoblasts of the pointed‐head worm are transplanted to the rounded‐head worm. (E) After the neoblasts have diffused, the head of the rounded‐head worm is amputated. Without a model of how target morphology is determined, it is impossible to predict what shape will regenerate, or if indeed regeneration will ever stop (given that neither set of neoblasts will be able to achieve their normal target morphology).

A fuller appreciation of the major gaps in our knowledge can be gained by taking an engineering approach: how would we build a robot that exhibited the observed repair and remodeling properties? It becomes immediately apparent that knowing the genes necessary for regeneration to occur (and their interactions) is not sufficient. A planarian body must recognize damage, and harness individual cells to rebuild exactly what is missing (in the right place, of the appropriate scale, and correctly oriented; Fig. 1A−A″). Most important, although not often discussed in the pursuit of *initiating* therapeutic regeneration, is how cells know when to stop. A crucial part of the planarian regenerative response is that each worm has a specific target morphology that, when achieved, causes rapid proliferation and remodeling to cease. A thought experiment (Fig. 1B−E) reveals the fact that, despite recent molecular insights, none of our models really address how target morphology is specified to make (even incorrect) predictions about situations in which this property is directly challenged. Importantly, these issues have implications well beyond planaria, as understanding the relationship between cellular growth control and large‐scale anatomical metrics is central to mammalian regeneration, cancer, and bioengineering of synthetic constructs (Doursat et al. 2013; Lobo et al. 2014b).

Thus, we take an information processing perspective to reverse engineering the planarian's robust pattern memory (Lobo et al. 2012) and abstract from the details of molecular pathways to ask: what control algorithms do individual cells and cell networks use to correctly integrate their activities toward a complex outcome with high reproducibility? What information is stored, processed, and communicated among which regions, to drive correct dynamical patterning and termination of regeneration? What measurements are being made in this system (size, shape, topology) and at what scales to enable precise coordination of three‐dimensional anatomy? What head shape will hybrid bodies make? How many heads will appear on circular fragments (one continuous wound all around the periphery) in worms treated with various reagents? Answers to these questions are not available from existing data, because we still largely lack the conceptual tools to integrate genetic and functional data into multi‐scale models that describe not only pathways but emergent patterning decision‐making. However, a number of recent studies shed light on aspects of this long‐term goal.

**Figure 2:**
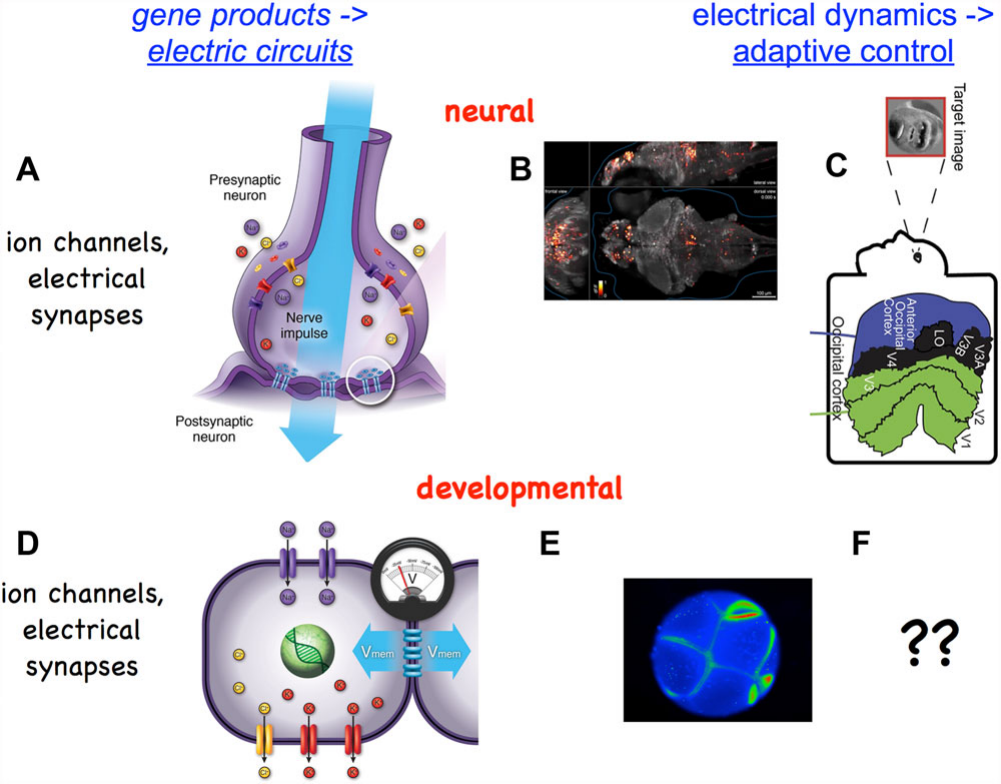
Bioelectric circuit elements in the brain and body. Gene loci encode ion channels and gap junction proteins (electrical synapses). However, it is the electrical dynamics of the resulting real‐time electric circuits that determine behavior of cells and tissues. In the brain, ion channels and electrical synapses (A) give rise to electrical activity in the brain (B) that integrates data, implements goal‐directed decision‐making behavior, and stores representations of geometric patterns. (C) On‐going efforts in a number of labs have shown that cognitive semantics of electrical brain states can be mathematically analyzed, such as computational pipelines that reveal which image a subject is visualizing mentally. In a highly parallel process, ion channels and gap junctions in non‐excitable cells (D) give rise to developmental bioelectricity―spatial and temporal patterns of resting potential occurring during development and regeneration (E). The ongoing effort to understand how developmental patterns are encoded in bioelectric properties (F) is the goal of cracking the bioelectric code. Images in panels A, D were made by Jeremy Guay of Peregrine Creative. Panel B used with permission from Ahrens et al., 2013. Panel C used with permission from Naselaris et al., 2009.

Here, we first briefly review what is known about the biochemical cues that regulate patterning. Then, we focus on recent data involving several novel pathways that are beginning to provide experimental access to questions about target morphology and large‐scale pattern control: ion channels, gap junctions (GJs) (electrical synapses), and neurotransmitters (Fig. 2). We discuss the potential implications of the fact that the prodigious information processing required to implement the planarian's feats make use of precisely the same basic molecular components by which brains implement memory, decision‐making, and distributed control of biochemical events to the service of higher‐level goal states. Finally, we discuss the need for generative, algorithmic models of patterning, and highlight recent efforts to harness computer science and artificial intelligence toward helping human scientists extract understanding from the ever‐growing deluge of data.

## Molecular genetic controls of planarian regeneration

The first step in any regenerative process is to detect that damage has occurred―that the current shape is not the correct shape. Within 30 min of cutting, a planarian initiates closure of the wound (Rink 2013) and begins regulation of the gene expression and physiological signaling required for proper patterning of the regenerating worm (Wenemoser et al. 2012). This wound response is potentially triggered by reactive oxygen species which burst within minutes of wounding (Pirotte et al. 2015), a damage signal conserved across regenerative organisms (Erler & Monaghan 2015). At 3 h *runt‐1* is expressed in neoblasts allowing for neoblast specification, and between 3 and 12 h genes expressing patterning factors are expressed subepidermally (Wenemoser et al. 2012). This gene expression is coupled with a wound‐specific, apoptotic response that occurs between 1 and 4 h (Pellettieri et al. 2010). Between 6 and 12 h, in the epidermis, extracellular matrix remodeling proteins are expressed, as well as an activator of the Notch signaling pathway (Wenemoser et al. 2012). At this time, *fst* is also expressed, which is required for the initiation of regeneration of lost tissue (Gaviño et al. 2013). Alongside the required gene expression, the regenerating planarian also experiences two peaks of mitotic activity, the first being across the entire body in response to injury and the second involving a recruitment of neoblasts to the wound site (Wenemoser & Reddien 2010). Once a blastema is formed, cells begin differentiation. The transition of the neoblasts to enter their differentiating state has been determined to be extracellular‐signal‐related kinase (ERK) dependent (Tasaki et al. 2011) and their differentiation according to axial polarity is regulated by epidermal growth factor receptor (Fraguas et al. 2014) and JNK signaling (Almuedo‐Castillo et al. 2014; Tejada‐Romero et al. 2015).

Subsequent control mechanisms begin determining neoblast fate (Umesono 2014) and the eventual tissue specification of stem cell progeny. After an amputation, neoblasts migrate to the wound site to develop a blastema through proliferation, which is, in part, regulated via PTEN through TOR signaling (Oviedo et al. 2008). SMG‐1 acts to keep growth in response to injury in check as mTOR signaling drives the process forward (Gonzalez‐Estevez et al. 2012). Some neoblasts express specific, lineage restricted transcription factors during regeneration and remodeling (Scimone et al. 2014), thus suggesting that neoblasts are specialized to a particular fate before they produce undifferentiated blastema cells with particular identities that they will adopt (Reddien 2013).

The new cells must then become appropriately arranged to establish correct polarity across all three of the body axes. Most of the work to date involves identifying the gene expression and signaling pathways necessary for this step, especially the Wnt signaling pathway. Knockdown of β‐catenin resulted in double‐headed or radial hypercephalized worms (Iglesias et al. 2008; Petersen & Reddien 2008) and upregulation of β‐catenin via APC‐1 and axin knockdown produced two‐tailed worms (Gurley et al. 2008; Iglesias et al. 2011). The bone morphogenetic protein signaling pathway largely mediates the dorsal/ventral axis as well as the dorsal planarian midline (Reddien et al. 2007) and, when disrupted, resulted in ectopic ventral sides of the planarian (Molina et al. 2007; Orii & Watanabe 2007). When proper axes are established via these signaling pathways, organs and structures need to be properly placed (Roberts‐Galbraith & Newmark 2015) and then in turn maintained homeostatically (Lin & Pearson 2014). Polarity determinants and those that pattern organ structures exhibit considerable crossover. For example, *PBX/extradenticle* is required for the proper patterning of several organ structures, but is also involved in the regulation of polarity genes such as *notum* and *wnt1* (Blassberg et al. 2013).

A planarian constantly scales and remodels to maintain its appropriate morphology. Autophagy allows the animals to adapt to their environment in times of stress, regulated in part by *Gtdap‐1* (González‐Estévez et al. 2007), whether it is due to injury or starvation. Regeneration after amputation also requires re‐sizing and scaling according to the new size of the planarian fragment. Alongside the initial apoptotic response at the blastema induced by wounding, there is a second apoptotic response that occurs systemically 3 days post‐amputation (Pellettieri et al. 2010). This is thought to allow for the worm to allometrically re‐scale according to its new size after tissue loss and is mediated by JNK activation (Almuedo‐Castillo et al. 2014). Whether it is induced by injury or an environmental factor requiring adaptive change, the ability to remodel existing tissues is crucial for the planarians’ pattern homeostasis.

## Endogenous bioelectric controls of planarian regeneration

It is clear that molecular details of cell signaling and stem cell specification are becoming ever more understood. How can we synthesize these reductive advances at the molecular level into predictive, systems‐level understanding of large‐scale planarian shape? Complexity, robustness, and emergence are some of the most difficult open problems in science today (Kauffman & Clayton 2006, Fernandez et al. 2013; Hoel et al. 2013). Fortunately, we have a possible roadmap: neuroscience has long faced the issue of functionally linking higher, emergent levels of system control to molecular pathways.

### Hypothesis: taking a cue from brain function

**Figure 3:**
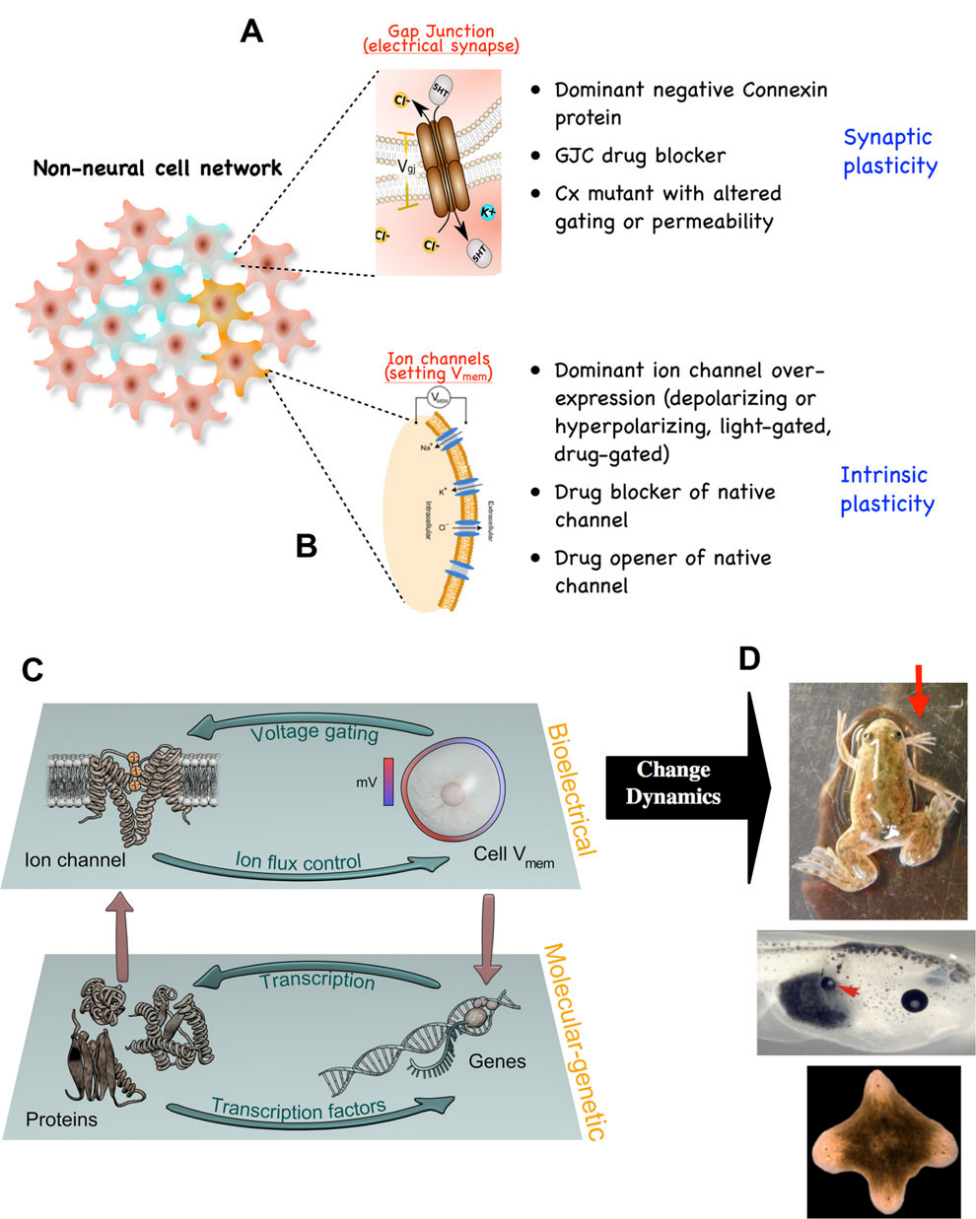
Bioelectric circuits―components, techniques, and functional roles. As in the brain, cells throughout the body form bioelectric networks. These networks have two main components: (A) electrical synapses (gap junctions), which allow neighboring cells to share bioelectric state, and (B) ion channels, which directly set the resting potential of specific cells based on a number of internal and external conditions. Modifying gap junctional connectivity within the network amounts to synaptic plasticity―editing the topology of bioelectrical connections. Importantly, gap junctions are themselves an important element of experience‐dependent plasticity in the central nervous system and perhaps in other contexts. In contrast, modifying cells’ bioelectric properties directly is akin to modifying the intrinsic plasticity of neurons. Both of these kinds of changes can now be induced by pharmacological and molecular genetic reagents, using targeted expression (and ligand gating) of wild‐type, constitutively active, and dominant negative ion channel and connexin proteins. Because some ion channels are themselves voltage‐sensitive, their activity can implement feedback loops between physiological parameters (voltage) and the activity of channel proteins. These feedback loops form an independent layer of control (C), which is functionally coupled to the layer implemented by canonical transcriptional networks (since bioelectric networks are implemented by ion channel genes, but themselves alter transcription of numerous loci). The information stored and processed in this layer is instructive and important for pattern formation (D), since experimentally modifying these dynamics has been shown to cause coherent, large‐scale patterning changes in vertebrate and invertebrate models, including production of limbs, eyes, and heads at ectopic locations.

Most of the work in regeneration is focused on biochemical signals, such as secreted molecules and transcriptional networks. However, a number of classical studies in planaria have examined the role of biophysical signals in this process—in particular, those mediated by electrical forces (Hyman 1932; Marsh & Beams 1952; Bonaventure 1957; Lange & Steele 1978). More recent work on the instructive patterning roles of bioelectrical gradients in vertebrate regeneration, development, and cancer (reviewed in Stewart et al. 2007; Adams 2008; Adams & Levin 2013; Levin 2014b; Mustard & Levin 2014) has made use of molecular physiology to study the sources, consequences, and transduction mechanisms of endogenous ionic signaling in non‐excitable tissues (Fig. 3). This work is now being extended to planaria (Barghouth et al. 2015). The information processing capabilities of the planarian body exhibit robustness, distributed (long‐range) integration, decision‐making, and cellular activity that drives changes until the correct anatomical end‐state is reached. These properties suggested investigating possible roles of pathways used by the CNS to perform similar functions (e.g., memory and goal‐directed activity) (Pezzulo & Levin 2015). In the brain, adaptive, flexible programs are implemented by electrical circuits consisting of GJs, ion channels, and neurotransmitters.

Interestingly, ion channels and the resulting bioelectrical gradients are evolutionarily far older than brains (Borgens 1982; Levin et al. 2006). Cells were using this versatile medium for communication and coupling physiology to cell behavior long before nervous systems evolved to optimize them for fast muscle control during behavior (Keijzer et al. 2013; Gao et al. 2015; Prindle et al. 2015). A now classic body of work in this field (Jaffe 1981; Nuccitelli et al. 1986; Borgens et al. 1989; Pullar 2011) has been augmented by modern molecular approaches in the last two decades showing that trans‐epithelial electric fields (Robinson & Messerli 1996; Yamashita 2013), ion currents (Smith & Trimarchi 2001; Reid & Zhao 2011), and resting potential gradients (Levin et al. 2002; Adams et al. 2006, 2016) within many tissue types serve as instructive signals for pattern regulation. Similar bioelectrical regulatory mechanisms have been shown in mammalian systems, both in vivo (Zhao et al. 2006; Lange et al. 2011; Leppik et al. 2015) and in vitro (Sundelacruz et al. 2008, 2013a, 2013b), and in a plethora of studies on aquatic vertebrate model species in the contexts of wound healing (Kucerova et al. 2011; Sebastian et al. 2015; Zhang & Bei 2015), regeneration (Adams et al. 2007, 2013; Monteiro et al. 2014), development (Dahal et al. 2012; Pai et al. 2012, 2015a; Perathoner et al. 2014; Lobikin et al. 2015b), and cancer (Chernet & Levin, 2013a, 2013b, 2014; Yang & Brackenbury 2013; Djamgoz et al. 2014). The intrinsic dynamics of bioelectric signaling (Cervera et al. 2014, 2015; Law & Levin 2015), the transduction machinery that allows voltage changes and ion flows to regulate gene expression and chromatin states (Carneiro et al. 2011; Tseng & Levin 2012a), and the downstream transcriptional targets of electrically mediated signaling (Pai et al. 2015b) have been extensively reviewed (McCaig et al. 2005; Stewart et al. 2007; Sundelacruz et al. 2009; Funk 2013; Bates 2015). Interestingly, the transduction of bioelectric states into downstream second messenger and nuclear events often involves neurotransmitters such as serotonin (Fukumoto et al. 2005b; Blackiston et al. 2011, 2015a; Lobikin et al. 2015a), but can also occur via clustering of RAS molecules (Zhou et al. 2015) and voltage‐sensitive phosphatases (Murata et al. 2005; Okamura & Dixon 2011). This biophysical layer of control not only guides individual cell functions like migration and differentiation, but also allows cellular networks to communicate and process global information for large‐scale patterning (Adams & Levin 2013; Levin 2013, 2014a, 2014; Mustard & Levin 2014). While endogenous electric fields provide long‐range guidance cues for a variety of galvanotactic cell types (Kucerova et al. 2011; Meng et al. 2011; Reid et al. 2011; Sun et al. 2013; Ozkucur et al. 2014), spatiotemporal patterns of resting potential regulate mitosis and differentiation (Cone & Cone 1976; Sundelacruz et al. 2008).

**Figure 4:**
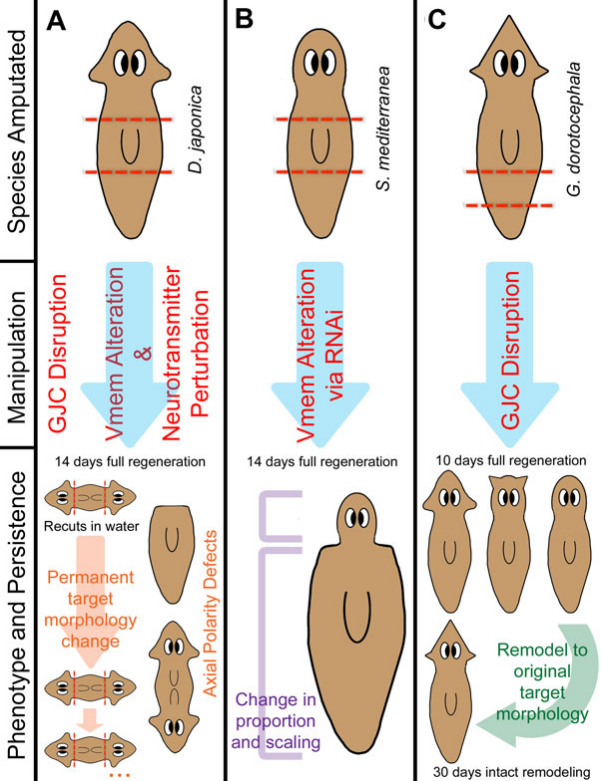
Regeneration pattern phenotypes revealed by perturbation of bioelectric networks: voltage gradients, gap junctional connectivity, and neurotransmitter signaling. Perturbing voltage gradients, gap junctional communication (GJC) and signals from neurotransmitters results in wide‐scale changes to polarity, shape, size, and scaling of varying permanence across different species of planaria. Since introduction of ectopic channels is not possible in planaria, modulation of bioelectric states is performed via RNAi‐mediated knockdown and pharmacological reagents that open or close electrogenic proteins. (A) *Dugesia japonica*, upon transient gap junction blockade, exhibits a permanent target morphology change to a double‐headed phenotype. When re‐cut in water, animals continue to regenerate a two‐headed morphology in future generations, long after the GJC inhibitor has left the worms’ tissues (Oviedo et al. 2010). Similarly, when exposed to the chloride channel opener ivermectin with depolarizing levels of chloride in solution, a double‐headed phenotype is again induced (Beane et al. 2011). Upon hyperpolarization with SCH28080, an H,K‐ATPase inhibitor, a headless phenotype is induced (Beane et al. 2011). Both the headless and double‐headed phenotypes can be induced by exposure to drugs that perturb serotonergic, dopaminergic, and cholinergic neurotransmitter pathways. (B) *Schmidtea mediterranea*, upon hyperpolarization via *H,K‐ATPase* RNAi, are unable to correctly scale and remodel proper proportion and size and therefore develop a shrunken head phenotype. Regenerated mature structures consist only of new tissue due to an inability to remodel old tissues (Beane et al. 2013). (C) *Girardia dorotocephala*, upon transient gap junction blockade, regenerate heads with characteristic head shapes (as well as brain shape and neoblast distribution) of other planarian species, including *D. japonica*, *P. felina*, and *S. mediterranea* (depicted left to right). After 30 days, these worms undergo intact remodeling to revert to the natural *G. dorotocephala* head shape (Emmons‐Bell et al. 2015).

Together, these diverse modes of ionic signaling enable organ‐level control, setting size of appendages (Perathoner et al. 2014) and programming identity of tissues, such as converting gut tissues into an eye with a bioelectrical perturbation (Pai et al. 2012). Recently, we hypothesized that these neural‐like mechanisms and strategies are conserved―utilized by the planarian body in making decisions about growth and form (Pezzulo & Levin 2015). This hypothesis makes a number of predictions, which have been tested by recent work that has implicated all of these bioelectric signaling mechanisms in the regulation of global pattern in planaria (Fig. 4).

### Prediction 1: Neurotransmitters regulate form, not only behavior

The first prediction of the brain analogy is the involvement of neurotransmitters in pattern regulation (not only in the control of behavior). Neurotransmitters and their receptors are widely present in flatworms (Ribeiro et al. 2005), and surveys have revealed numerous effects of neurotransmitter modulation on planarian regeneration (Villar & Schaeffer 1993). Indeed, recent work has shown that the dynamics of neurotransmitter signaling can alter regenerative morphology. A key study that was originally designed to develop sophisticated anti‐parasitic therapeutics now has implicated neurotransmitters as important factors in the regulation of polarity. Praziquantel, an anti‐parasitic, synthetic compound which increases the permeability of the tegument to Ca^2+^, induced bipolar phenotypes in planaria (Chan et al. 2014) and can be rescued with exogenous serotonin (Chan et al. 2015). A broad drug screen designed to identify other potential anti‐parasitic drugs like praziquantel also screened for patterning defects and found cholinergic, dopaminergic, and serotonergic drugs all induced changes in planarian regenerative polarity (Chan et al. 2014). This included but was not limited to pharmaceuticals that are commonly prescribed to human patients, such as the selective serotonin re‐uptake inhibitor fluoxetine, which induced a headless phenotype, and the antipsychotic dopamine antagonist haloperidol, which induced both double‐headed and headless phenotypes (Fig. 4A).

Serotonin signaling has been implicated in regulating cell activity and pattern in embryonic left−right asymmetry (Fukumoto et al. 2005a, 2005b), neural growth (Blackiston et al. 2015a), and metastasis (Blackiston et al. 2011). Interestingly, serotonin has been shown to regulate gap junctional communication (GJC) (Rorig & Sutor 1996; Orellana et al. 2013) whilst also being a GJ permeable morphogen (Levin 2006; Gairhe et al. 2012), which suggests the possibility of feedback loops with rich and complex behavior. Thus, the voltage−serotonin control cycle may function beyond the CNS, forming a second messenger pathway for the control of intracellular events by bioelectric signaling. With better techniques to help visualize cellular networks such as photo‐uncaging (Li & Zheng 2012; Rea et al. 2013) and neurotransmitter reporters (Balaconis & Clark 2012), exciting future discoveries no doubt await the characterization of neurotransmitter activity throughout the planarian regenerative process.

### Prediction 2: Bioelectric circuits determine anterior−posterior polarity and morphallaxis

In the CNS, the function of bioelectric circuits triggers recall of specific compound memories and complex (modular) behaviors (Maisto et al. 2015). The brain offers a paradigm of how cellular signaling gives rise to integrated decision‐making and goal‐driven activity at the whole organism level. Thus, a second prediction is that modulation of ion‐channel‐dependent bioelectric states during regeneration should be able to specifically alter coherent (large‐scale) properties, such as anatomical re‐specification and scaling. An emerging theme in developmental bioelectricity studies is that, while cell migration is guided by electric fields (McCaig et al. 2005), anatomical specification appears to be determined in part by the spatial distribution of resting potentials of cells, *V*_mem_ (Levin 2012a; Levin & Stevenson 2012), as has been shown for pre‐patterning of the face (Vandenberg et al. 2011) and brain (Pai et al. 2015a) and for induction of eyes (Pai et al. 2012) in vertebrate models. Involvement of bioelectric signaling in regeneration is reviewed by Levin (2009) and Stewart et al. (2007).

#### Anterior−posterior polarity

An inverse drug screen implicated the H,K‐ATPase in AP patterning; it was subsequently shown to underlie the planarian endogenous bioelectric gradient (Beane et al. 2011). This process appears to be conserved, since the H,K‐ATPase also regulates *V*_mem_ in mammals for proper functionality of the gut and cochlea (Shibata et al. 2006; Kaufhold et al. 2008), biomineralization of the developing sea urchin skeleton (Schatzberg et al. 2015), and left−right patterning in embryonic frog, sea urchin, and chick (Levin et al. 2002; Duboc et al. 2005; Hibino et al. 2006; Aw et al. 2008; Morokuma et al. 2008).

Using the specific inhibitor of H,K‐ATPase, SCH28080 (SCH), a wide‐scale, relatively hyperpolarized bioelectric state across entire regenerating *Dugesia japonica* fragments was induced, eliminating the normal relative depolarization at the anterior blastema. When treated within the first 72 h of regeneration, this abolished formation of anterior structures and expression of anterior genes, and resulted in headless, one‐tailed phenotypes (Fig. 4A) (Beane et al. 2011), which are similar but not identical to those produced via perturbation of the Wnt pathway which produced headless, two‐tailed worms (Gurley et al. 2008; Iglesias et al. 2011). Investigation of epistasis between bioelectric control of patterning and known genetic elements showed that H,K‐ATPase inhibition blocked ectopic heads (Beane et al. 2011) normally formed by β‐catenin RNA interference (Gurley et al. 2008; Petersen & Reddien 2008). Thus, the details of the interactions between bioelectric gradients and canonical biochemical signaling pathways have begun to emerge. However, considering the bioelectric state as being entirely downstream of Wnt signaling is not the whole story. H,K‐ATPase function is also required to clear Wnt11 expression from anterior regions (Beane et al. 2013), suggesting a feedback loop between *V*_mem_ and Wnt signaling. A similar bi‐directional loop has been described between Notch signaling and *V*_mem_ gradients in vertebrate brain development (Pai et al. 2015a). Importantly, the suppression of genetically induced malformations (β‐catenin two heads or Notch‐mutation‐induced brain defects in frog) (Pai et al. 2015a) by exposure to drugs already approved for human use is a proof‐of‐principle for the strategic use of ion channel drugs (so‐called electroceuticals) to address mispatterning during birth defects (Masotti et al. 2015; Adams et al. 2016), regenerative repair (Tseng et al. 2010), and cancer (Arcangeli et al. 2009; Gupta et al. 2009; House et al. 2010; Lobikin et al. 2012; Chernet & Levin 2013b; Villanueva et al. 2015).

*V*_mem_ patterns can be manipulated readily, using many pharmacological reagents under the guidance of the Goldman equation; this is particularly useful in planaria, where drug‐induced ion channel opening is a convenient method for gain‐of‐function perturbation. Ivermectin drives changes in *V*_mem_ by activating glutamate‐gated chloride (GluCl) channels (Shan et al. 2001) and can depolarize or hyperpolarize cells based on the concentration of extracellular chloride. Manipulating *V*_mem_ using ivermectin has been shown to induce regeneration in non‐regenerative (refractory age) tail stumps in *Xenopus* (Tseng & Levin 2012a), and to promote head regeneration in non‐regenerative fragments of *Macrostomum lignano* (Simanov et al. 2012). In *Dugesia japonica* flatworms, ivermectin exposure created a wide‐scale depolarization across regenerating fragments with two regions of high relative depolarization at both anterior and posterior blastemas. Upon regeneration, posterior blastemas were anteriorized, resulting in mature worms with a double‐headed phenotype (Fig. 4A). Raising external chloride to hyperpolarize the worms blocked ectopic anteriorization, suggesting that *V*_mem_ changes, rather than some other function of GluCl or an off‐target effect of ivermectin, are the key early step in determining AP polarity in regenerating planaria (Beane et al. 2011).

#### Head shape and scaling

Advancements in regenerative medicine will require induction of not only correct anatomical identity of new structures, but also their correct size and proportion. Planaria have the striking ability to allometrically scale their organs and tissues in order to maintain their proper proportions after regeneration (Oviedo et al. 2003). Although many aspects of morphallaxis (Morgan 1901) remain mysterious, recent findings in planaria suggest that *V*_mem_ plays a role in these processes, as it does in size control in vertebrates (Perathoner et al. 2014). Upon *H,K‐ATPase* knockdown, worms lost their relative depolarization at the anterior blastema and became relatively hyperpolarized across the entire fragment, causing the regeneration of small shrunken heads (Fig. 4B) with incomplete neural tissue and reduced expression of anterior genes (Beane et al. 2013). Correspondingly, expression of posterior genes extended ectopically toward the anterior region of the worm; moreover, the size and location of the pharynx was unable to scale according to the new size of the worm, and any anterior tissues were made using new tissue rather than remodeling the old, suggesting a failure to execute proper morphallaxis. Regeneration in a variety of systems requires some level of remodeling or dedifferentiation to successfully promote the regenerative process (Jopling et al. 2011). If the process of remodeling old tissue to make way for new tissue can be induced by simple manipulation of ion transporters, regeneration in currently non‐regenerative tissues may be more accessible in the future. What cell‐level behaviors mediate the ability of bioelectric gradients in coordinating cell growth across three dimensions?

Regeneration in many species involves an apoptotic response (Ryoo & Bergmann 2012), including in planaria (Hwang et al. 2004; Almuedo‐Castillo et al. 2014), allowing for proper size and scaling to maintain proportion. Indeed, regeneration requires a correct amount of apoptosis (Tseng et al. 2007; Chera et al. 2009). Planarians experience two waves of apoptosis during regeneration, the first being specifically at the wound site 1−4 days post‐amputation (dpa) and the second being at 3 dpa which occurs throughout the animal (Pellettieri et al. 2010). Inhibiting apoptosis in planaria with M50054 (Tsuda et al. 2001) resulted in regenerates with relatively small heads and large pharynxes, a phenotype very similar to that seen with H,K‐ATPase inhibition (Beane et al. 2013). Consistently, after *Smed‐H,K‐ATPase* RNA interference, the second wave of apoptosis at 3 dpa to drive remodeling essential for proper regeneration was inhibited. This implies that the role of bioelectric signaling in planarian remodeling is specifically involved in the apoptotic response that is necessary for proper size, shape, and proportion of tissues after regeneration.

#### Species‐specific head morphology

Alongside organ identity and scaling, correct shape is also crucial for successful regeneration. Since bioelectric distributions guide and pre‐pattern the morphology of complex structures such as the face and brain (Vandenberg et al. 2011; Pai et al. 2012), it has been shown that direct modulation of voltage states in vivo can alter morphogenesis via the control of numerous downstream patterning genes (Pai & Martyniuk 2015). However, bioelectric circuits operate within cells connected by electrical synapses, also known as GJs (Cooper 1984; Palacios‐Prado & Bukauskas 2012). Recently, we asked two questions: (1) could modification of overall bioelectric network connectivity give rise to coherent patterning changes during regeneration, and (2) is it possible to obtain evolutionarily relevant patterns. The results suggested that shifting among different regions of planarian morphospace (Stone 1997) is possible by physiological perturbation alone (Emmons‐Bell et al. 2015).

When GJC was disrupted in *Girardia dorotocephala* (GD), it induced a finer‐grain *V*_mem_ regionalization among the endogenous bioelectric network and worms regenerated heads with an altered shape morphology that quantitatively resembles that of multiple other flatworm species (Fig. 4C) (Emmons‐Bell et al. 2015). This resemblance is more than skin deep: the external shape of the head not only was converted to the shapes of distant planarian relatives, but also was extended to changes to other species‐specific brain morphologies and neoblast distributions. The exact same treatment of a cohort of GD worms produced four types of worm heads in characteristic frequencies (proportional to their evolutionary distance from GD). It is not yet known whether this stochastic property is a consequence of the still relatively crude method of network topology perturbation, or whether it is an intrinsic aspect of the dynamics of this system. The ability to induce a different species’ head shape in a genetically wild‐type worm is fascinating, and suggests that the bioelectric network is a profound regulator of species‐specific morphology. It remains to be seen whether changes in the dynamics of bioelectrical circuits have been widely exploited by evolution to explore variations of anatomical structure.

**Figure 5:**
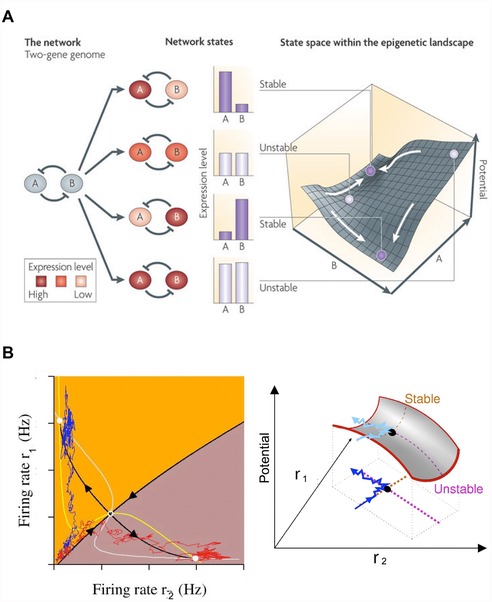
Familiar networks and attractors that underlie stable behavior in complex systems. Dynamic systems theory can be used to study how biochemical circuits achieve specific and distinct stable states. (A) A property of cross‐inhibitory gene circuits is a bistable potential landscape with two stable states and an intermediate saddle point of instability. Knowledge of the gene expression levels maps to a given point on the potential landscape, and the final cell can end in one of two basins as a direct result of the initial expression level of the two genes (image used with permission from Fig. 2 in Brock et al. 2009). (B) The firing rate of neurons in neural computational circuits can be mapped to stable states of decision‐making (image used with permission from Fig. 4 in Wong 2008). Two “trials” for a neural network are transformed into a phase space as traced by the red and blue lines. As with (A), the neural circuit produces a bistable network with a central saddle point (Wong and Huk 2008). The intersection of the white and yellow lines (nullclines) give the system's two steady‐states. The orange and brown indicate boundaries where the network's initial starting conditions would lead to a particular basin of attraction. Based on their initial firing rates, both of the trials would end in the orange attractor steady‐state in a system without noise. However, due to system noise in firing pattern, the red trial ends up in the brown attractor, demonstrating how small disturbances in complex systems can cause large‐scale changes to final decision‐making.

**Figure 6:**
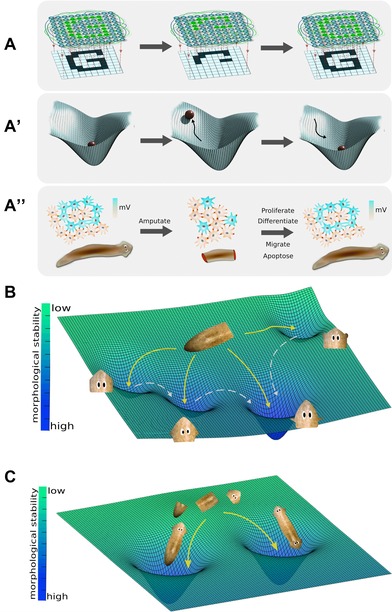
Visualizing planarian regeneration as an energy landscape within a morphospace. One way to visualize planarian regeneration is (A) as the function of a large network of electrically coupled cells. Such networks have been shown (in computational neuroscience and artificial intelligence research) to have a planaria‐like property of holographic memory storage: a trained network can recreate the entire pattern despite deletions of the pattern or of the network components. A well‐accepted mathematical paradigm for understanding the global properties of such networks (A′) is as an energy landscape, with attractors corresponding to specific stable modes of the network. In our analogy, amputation raises the energy of the system, temporarily pulling it out of the attractor to which the system tends to return. One hypothesis (A″) is that these networks are responsible for storing the pattern of a normal planarian and, when damaged, issuing cell‐level commands (differentiate, proliferate, and other instructions) that restore the anatomy (in parallel to how recall of complex geometric memories can be triggered by stimuli and induce goal‐directed behavior in cognitive science studies of animal behavior). This hypothesis makes a prediction: that coherent changes in patterning will result from experimentally induced changes of the bioelectric network's topology or dynamics. (B) Indeed, it has been shown (Emmons‐Bell et al. 2015) that altering the bioelectric connectivity in *Girardia dorotocephala* results in regeneration of one of four discrete head types. On this view, amputation of head and tail causes the system to move to an unstable state from its basin of attraction. Partial interruption of gap junction communication between cells (reduced connectivity and thus altered dynamics of the network) induces head wounds to regenerate (yellow arrows) new heads that resemble closely related flatworm species that are regions of stability in the regeneration morphospace landscape (left to right: *Schmidtea mediterranea, Dugesia japonica, and Polycelis felina*) as well as heads of the original species in the center basin. The probability of regenerating a certain head shape is proportional to the evolutionary distance from *Girardia dorotocephala*. These states are non‐permanent (shallow basins of attraction) and over time will remodel into their final morphological state (white dashed arrows) to the deepest and most stable basin of attraction of the original head shape. (C) A similar experiment in *Dugesia japonica* induces a new attractor that is as stable as the wild‐type worm morphology. As the processes of regeneration act to minimize the free energy of the system, the trunk piece can shift to an attractor that corresponds to bioelectric circuit modes whose activity induces a double‐head phenotype (right basin). Upon further amputation of double‐headed planaria, the phenotype persists, demonstrating that this is a stable morphological state. Graphics in Panel A were made by Alexis Pietak.

How to infer the large‐scale outcomes (which kind of head, how many heads, etc.) from cell‐level properties and signals? This question has been addressed for gene regulatory networks (Fig. 5A), using dynamical state spaces built to describe transcriptional circuits that have been used to map complex system behavior (Huang et al. 2009; Huang 2011; Halley et al. 2012). More directly relevant to physiological networks, similar approaches (Fig. 5B) have been used to understand global behaviors of electrical activity in neural networks during decision‐making (Wong & Wang 2006). Importantly, in planarian regeneration, as in the brain, circuit dynamics are not directly revealed from ion channel expression data but are complex and nonlinear. Such dynamics must be modeled quantitatively to understand their emergent properties (Cervera et al. 2015; Law & Levin 2015). One possibility is that different anatomical outcomes correspond to specific attractors in the dynamical state space of the bioelectric network formed by the planarian body; in this paradigm, bioelectric perturbations can shift the system from a default (genome‐specified) attractor to another nearby one (Fig. 6). We are currently working to computationally model this process, to quantitatively map stable attractors to underlying physiological details, and thus to gain more control over the resulting shapes.

#### Transduction mechanisms: a focus on calcium signaling

How do bioelectrical events become transduced into transcriptional readouts and chromatin modification? In the brain, this job is performed by calcium signaling: calcium channels and a set of calcium‐sensitive receptor pathways convert electrical activity into biochemical and genetic responses. Not surprisingly, voltage‐gated calcium channels (Ca_V_) have been shown to transduce *V*_mem_ signals into growth cone attraction in *Xenopus* (Nishiyama et al. 2008), transcriptional patterning in *Xenopus* by instructing the development of the eye (Pai et al. 2012), and the development and maturation of mammalian neurons (Nakanishi & Okazawa 2006). In planaria, similar to the phenotype observed by treatment with ivermectin, treatment with praziquantel, a Ca_V_ activator, resulted in a double‐headed phenotype upon regeneration (Nogi et al. 2009; Chan et al. 2013). Analogously, the Ca_V_ inhibitor nicardipine induced a headless phenotype. Normally, calcium is upregulated in anterior blastemas; this became disrupted upon hyperpolarization of regenerating fragments with H,K‐ATPase inhibitors. These data suggest that calcium signaling could be involved in translating *V*_mem_ signals into anterior gene expression in planaria (Beane et al. 2011) probably involving Hedgehog signaling for proper cell differentiation (Zhang et al. 2011). Ever‐finer dissection of the mechanistic details of voltage transduction on a single cell level complements efforts to synthesize signaling into large‐scale patterning behavior.

### Prediction 3: Electrical synapses underlie morphological plasticity and pattern memory

The information required to determine anatomical identity is not local: upon bisection, the two pieces' blastemas must make a head or a tail respectively, despite the fact that the wound cells were adjacent neighbors before the cut and thus at the same location in the worm. The same positional information gives rise to two distinct anatomical outcomes, based on the cells’ context (the rest of the fragment). Thus, knowing local (positional) information is not sufficient―a blastema needs cues from the rest of the body (Where am I located? Which way am I facing? What else already exists in the fragment and does not need to be re‐created?). Targeting mechanisms that can underlie such long‐range tissue coordination, our laboratory examined the role of GJs (Levin 2007; Yamashita 2010) in planarian regeneration (Nogi & Levin 2005).

GJ proteins are conductive channels within the cell membrane that allow for intercellular communication and signaling via ions and very small molecules (Phelan 2005; Scemes et al. 2007). Signaling mediated by GJs has been shown to support the proliferative abilities of both embryonic and somatic stem cells (Wong et al. 2008), including planaria (Oviedo & Levin 2007). GJ genes (innexins and connexins) are widely expressed during development, establishing electrically‐isopotential cell compartments (Lo & Gilula 1979; Pitts et al. 1988). GJs are required for the physiological maintenance of many mammalian tissues (Maeda & Tsukihara 2011), regeneration of retina (Umino & Saito 2002) and zebrafish fins (Hoptak‐Solga et al. 2008), and patterning of the left−right axis (Levin & Mercola 1998, 1999; Chuang et al. 2007).

Invertebrates form functional GJ channels using innexins (Phelan et al. 1998). Innexins are expressed throughout the planarian and are classified into three functional groups as determined by their expression pattern: the first in the gut, the second in the nervous system or blastema, and the third in the parenchyma or protonephridia (Nogi & Levin 2005). When GJC was inhibited pharmacologically (Nogi & Levin 2005) or via RNAi (Oviedo et al. 2010) in *D. japonica*, a re‐specification of AP polarity occurred and viable two‐headed planaria resulted (Fig. 4A). Interesting differences reveal themselves when this outcome is compared to the above‐described alteration of head shape in GD worms.

In GDs, the regeneration of other species’ heads is a two‐phase process: the heads regenerate with new shapes in the normal timeframe (<10 days), but within the next 30 days they remodel back to a GD‐specific shape (Fig. 4C). This is strikingly similar to what happens in salamanders when a tail blastema is grafted to the flank: initially, a tail grows, but some months later it remodels into a limb―a structure more appropriate to its new global position (Farinella‐Ferruzza 1953, 1956). This temporary shift into a different stable attractor by GJ (electrical synapse) somatic networks has a clear parallel in neural networks: attractors in state space of neural networks represent individual memories (Fuster 1998; Wills et al. 2005). Depending on the strength of the attractor state, memories can have different degrees of permanence.

The middle third fragments from two‐head animals, derived from GJC inhibition, continue to regenerate as two‐headed in subsequent amputations without any further GJ blockade (Fig. 6B). Likewise, two‐head animals result when just one of the heads is removed in plain water from a two‐head worm. This is permanent, months after the initial GJ blocker exposure (which was shown to leave tissues within 24–48 h) (Oviedo et al. 2010). On the one hand, this is quite reasonable given that GJs are one of the ways plasticity (memory) is implemented in the CNS: GJs serve as versatile transistors, able to “freeze” transient physiological stimuli into stable, permanent changes of network topology (Palacios‐Prado & Bukauskas 2009; Pereda et al. 2013). On the other hand, it is remarkable that a brief, transient, physiological perturbation can permanently alter a complex metazoan's target morphology (the shape to which an animal regenerates, and the morphology that, once reached, signals an end to massive remodeling).

This two‐head permanence has many potential implications, not the least of which arises from the fact that it is stable across the animal's most common reproductive mode (fission) (unpublished observations). First, it impacts the relationship between genomic sequence and bodyplan structure. This would become sharply apparent if the two‐head worms were allowed to reproduce in the wild (assuming they could compete with wild‐type animals and survive). One can imagine wanting to sequence the genomes of one‐head and two‐head worms, looking for the mutations that drove this speciation event resulting in significant morphological diversity. The key difference here is not provided by the genomic sequence, and reminds us that real‐time physiomic profiling must be added to proteomics and genomics if we are to understand and predict large‐scale shape.

The second important aspect is the relationship to “epigenetics.” Bioelectric properties, and their permanence, are certainly a kind of epigenetics in the original full sense of the concept (Jablonka 2012). While it is possible that some aspect of chromatin modification (today's main focus of epigenetics) is involved, and indeed has been shown to be involved in neoblast regulation (Hubert et al. 2013; Robb & Sanchez Alvarado 2014), it must be kept in mind that the “reprogrammed” abnormal head blastema is discarded at each round of cutting (Oviedo et al. 2010). Only trunk fragments are taken, showing the holographic (distributed) nature of this pattern change: headless fragments from two‐head worms have been reprogrammed to work towards a two‐headed outcome upon regeneration (Fig. 4A, 6C). Thus, a truly explanatory model of this phenomenon will need not only to identify molecular targets *required* for two‐head persistence memory, but to explain how the proposed signaling is *sufficient* to specify the right number of heads for each fragment in each case. We are currently analyzing models of realistic bioelectric circuits that exhibit the necessary patterning and memory behavior. While editing of target morphology has been seen before in crabs and deer antlers (reviewed in Lobo et al. 2014b), it is clear that the planarian model system is by far the most molecularly tractable example and will greatly facilitate the study of pattern memory.

## Putting it all together: computational approaches

Ultimately, the building blocks of regeneration are sure to include biochemical, bioelectrical, and physical forces. Each will require an appropriate paradigm (gene regulatory networks, mechanical models of stresses and tensions, bioelectrical circuit dynamics and neural‐like networks). However, the final product must be not only a list of components required for regeneration to occur, nor even a highly detailed interactome or regulatory diagram. The ultimate end‐game for this field is the development of constructivist, algorithmic models that specify exactly what is going on at each step, and explain why these steps are sufficient to give rise to the correct shape from different starting conditions (the robust shape regulation that is observed in planaria). Such models can then be used to infer external modulations that can alter shape to desired outcomes or induce regenerative repair in biomedical settings. Modeling is also necessary because the stable and stochastic behaviors of chemical and physical pathways are often highly nonlinear and emergent. Here, we discuss recent efforts to glean insight into patterning homeostasis in planaria.

### Human scientists’ work on planarian patterning

**Figure 7:**
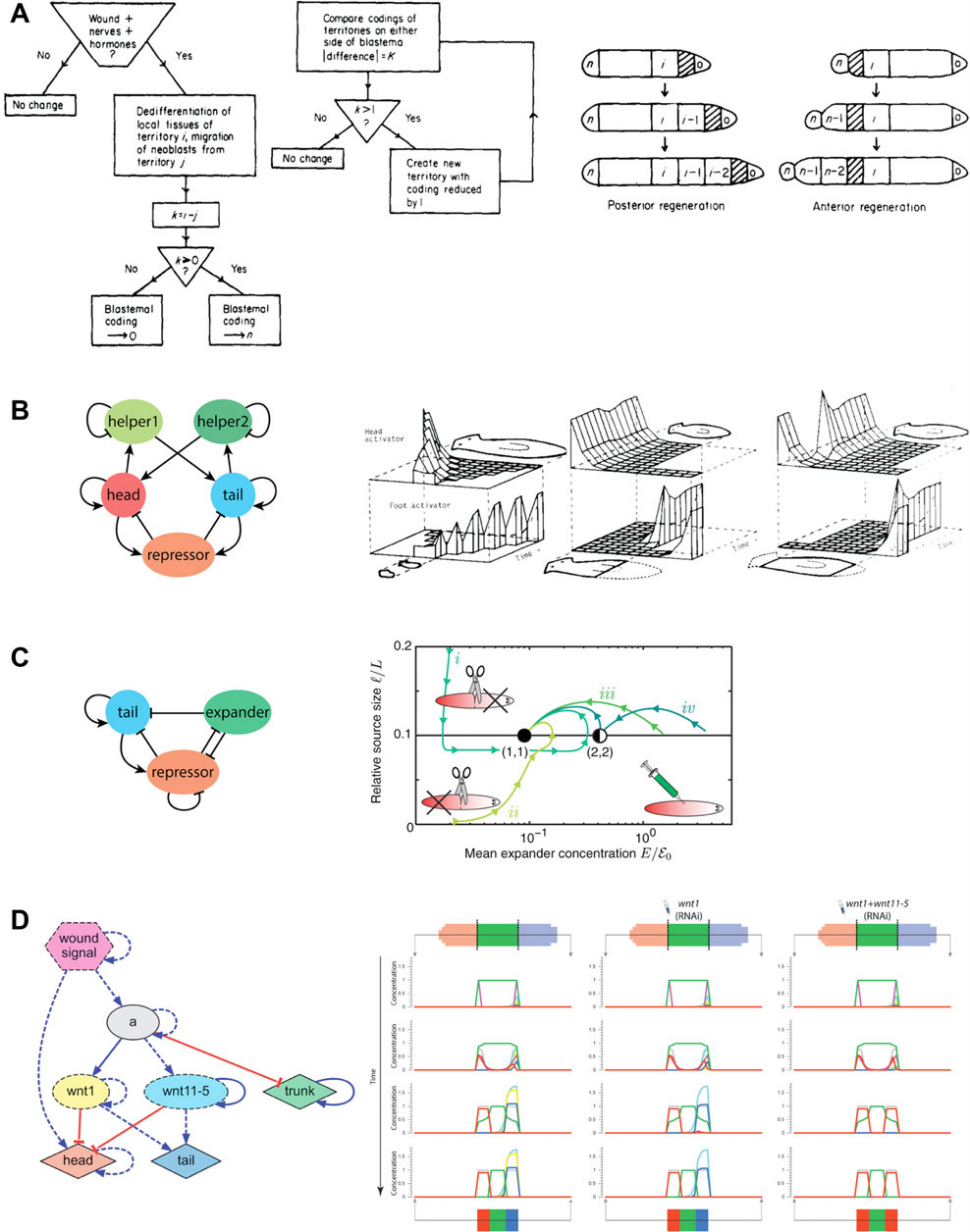
A selection of mechanistic models of planarian regeneration proposed in the literature. (A) The serial theory of regeneration based on the comparison and regeneration of sequentially coded territories (image used with permission from Slack 1980). (B) Dynamical chemical model based on local self‐activation and long‐range lateral inhibition (Meinhardt 1982). (C) A simplified dynamical model based on three diffusible molecules that can account for the scaling of the self‐organized head−tail planarian pattern (Werner et al. 2015). (D) A reverse engineered dynamic regulatory network able to recapitulate the resultant phenotypes of specific surgical, genetic, and pharmacological interventions (Lobo & Levin 2015).

In order to understand the mechanisms of planarian regeneration, mathematical and computational models have been proposed that allow us to mechanistically explain and predict the regenerative dynamics of the worm (Fig. 7). The first models proposed to explain planarian regeneration were descriptive. Morgan, inspired by the correlation between the regenerative capacity of flatworms and the AP location level of the amputation (Sivickis 1931; Brøndsted 1955), suggested the existence of a substance concentration gradient signaling the regeneration of a head versus a tail (Adell et al. 2010). This idea was further developed in a series of historical models, including Child's gradient model (Child 1941), Spemann's organizer concept (Spemann & Mangold 2001), and Wolpert's positional information theory (Wolpert 1969). Similar gradient models have been suggested for explaining planarian regeneration, including morphogen concentration gradients (Adell et al. 2010; Schiffmann 2011) and mitotic activity gradients (Oviedo & Levin 2007). However, these descriptive models based on gradients do not represent a mechanism for which a given tissue can decide to regenerate either a head or a tail: the cells on either side of a transversal cut through the middle of the worm will essentially have the same gradient or positional information, yet one side will regenerate a head while the other will regenerate a tail.

The first mechanistic model proposed for planaria was Slack's serial threshold theory of regeneration (Slack 1980). This algorithmic explanation proposed the existence of a discrete set of territories dividing the worm along the AP axis, each of them containing an ordered coded sequence. After an amputation, neoblasts from the remaining territories migrate towards the wound site and then compare their original territory code with the territory code at the wound site. If the wound site has a higher code than the neoblasts, then the blastema adopts the maximum possible code; otherwise it adopts the minimum possible code. Regeneration proceeds by restoring the tissues corresponding to those codes that should be located between the wound and this new code in the blastema. In this way, this model specifies step by step the mechanisms that are sufficient to restore the morphological patterning of the worm. In a similar fashion, the intercalary regeneration model (Agata et al. 2003) hypothesizes that each region of the worm has a positional value, which is then used in an injury site to establish which regions are missing. This model can explain the regeneration of all the intermediate structures between two joined worm pieces, even if one of them is inverted, which produces the duplication of existent structures (Santos 1929; Slack 1980). However, certain predictions of these models based on positional information do not agree with specific experiments, such as the classic observation of the regeneration of a new pharynx and other structures from the old tissue, instead of from the blastema (Morgan 1898).

Inspired by Turing's reaction−diffusion mechanisms of biological pattern formation (Turing 1952), Meinhardt and Gierer proposed dynamical chemical systems based on properties of local self‐activation and long‐range lateral inhibition that control the generation (and regeneration) of specific patterns from near‐homogeneous states or perturbations (Gierer & Meinhardt 1972; Meinhardt 1982; Meinhardt & Gierer 2000). These dynamic models based on the self‐ and cross‐regulation of diffusible chemical species can explain the maintenance of polarity, the correct re‐patterning, and the scaling ability of planarian worms after surgical manipulations (Meinhardt 2009). Interestingly, a classic local activation and lateral inhibition model extended with an extra third diffusible molecule can account for the scaling of the self‐organized head−tail pattern to precisely match the variable worm length (Werner et al. 2015). Specific molecular components have been proposed for these dynamic mechanisms, such as cAMP and ATP, which can diffuse through GJs (Schiffmann 2011). Reaction−diffusion models still lack specific details to account for many knockdown experiments resulting in abnormal morphologies, dorsal−ventral joining experiments, the regeneration of multiple AP axes, or the mechanisms of neoblast migration and differentiation.

In addition to diffusion due to differences in concentration, models based on bioelectricity have been proposed to explain the transmission of long‐range signals during planarian regeneration (Lange & Steele 1978). Inspired by the effect of external electrical fields to reverse the polarity of the worm (Dimmitt & Marsh 1952; Marsh & Beams 1952), a negatively charged substance inhibiting the regeneration of brain tissue has been suggested to be produced by the brain itself, which then will electrically diffuse due to the global planarian bioelectrical field: negatively charged in the anterior region and positively charged in the posterior region. In this way, an amputation removing the brain will result in the charged molecule disappearing, which will trigger the formation of new brain tissue specifically at the most anterior side due to its lower concentration caused by the remaining global bioelectric field. This bioelectric model can account for the regeneration of double heads, or complete change of polarity after the application of external electric fields, which at different intensities can cause the charged molecule to stop electro‐diffusing or reverse its direction, respectively (Marsh & Beams 1947). A diffusion model has also been proposed for the control of serotonergic signals by bioelectric gradients during left−right patterning (Esser et al. 2006; Levin et al. 2006; Zhang & Levin 2009).

### Reverse engineering planarian regeneration: an assist from artificial intelligence

Models based on the theory of dynamical systems and differential equations represent one of the most useful approaches for mechanistically describing biological regulation of shape and form (Jaeger & Sharpe 2014). However, formulating the precise differential equations that can recapitulate the dynamics and behaviors of a given biological phenomenon is a very difficult task (Lobo et al. 2012), and indeed represents an inverse problem with no analytical solutions (Lobo et al. 2014b). Instead, heuristic computational methods have been proposed for the automatic construction of dynamic models directly from experimental data (Yeung et al. 2002; Bonneau et al. 2006; Schmidt & Lipson 2009; Sirbu et al. 2010). The reverse engineering of the regulatory network controlling the early patterning of the *Drosophila* embryo from one‐dimensional gene expression data represents one of the most successful applications of these heuristic methods (Reinitz et al. 1995, 1998; Jaeger et al. 2004; Perkins et al. 2006; Manu et al. 2009; Crombach et al. 2012; Becker et al. 2013).

**Figure 8:**
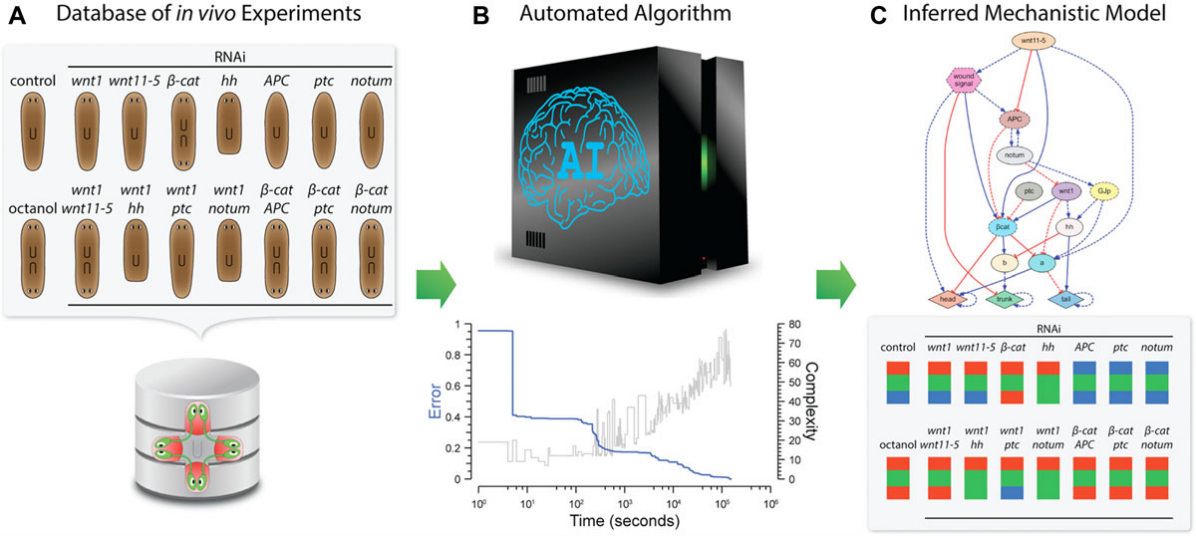
An automated computational method reverse engineered a dynamic regulatory network explaining the main experiments of planarian regeneration. (A) A formalized database of experiments using a novel mathematical planarian ontology was created to encode surgical, genetic, and pharmacological interventions and their resultant morphologies from the literature. (B) The algorithm, based on evolutionary computation, performs many in silico experiments to search for a dynamic regulatory network that can explain all the experiments in the input dataset. (C) This method inferred (Lobo & Levin 2015) the most comprehensive mechanistic model of planarian regeneration to date, able to recapitulate the most important head, trunk, and tail patterning experiments in the literature, as well as predict novel phenotypes and regulatory genes.

Recently, a novel heuristic computational method (Fig. 8) has been demonstrated for the reverse engineering of dynamic models of planarian regeneration patterning directly from resultant morphological perturbation experiments (Lobo & Levin 2015). The method takes as input a dataset of planarian experiments formalized with a specifically designed mathematical ontology (Lobo et al. 2013a, 2013b). Crucially, this formalization permits the unambiguous specification of precise surgical manipulations, genetic and pharmacological treatments and, using a mathematical graph representation (Lobo et al. 2011), the resultant planarian morphologies. The method then uses a whole‐organism simulator for testing the error of a given dynamic model with respect to the set of experiments formalized in the input dataset, scoring the models according to the level of similarity between the in vivo and in silico resultant morphologies. Based on the algorithmic techniques of evolutionary computation (Holland 1975), the method maintains a population of evolving models, iteratively crossing, mutating, and selecting the best ones for the next generation. When a model is found that can perfectly recapitulate all the experiments in the input dataset, the algorithm stops and the found model is returned.

This method was successfully validated by reverse engineering the most comprehensive dynamic model of planarian regeneration found to date (Lobo & Levin 2015). The input experimental dataset included the formalization of the most important head‐versus‐tail planarian experiments in the literature, including surgical, genetic, and pharmacological perturbations (Gurley et al. 2008; Iglesias et al. 2008; Petersen & Reddien 2008, 2009, 2011; Rink et al. 2009; Oviedo et al. 2010). After 42 h (using 256 cores in a computer cluster), the algorithm found a dynamic model (a set of differential equations) that, when simulated, could recapitulate all the experiments included in the input dataset. Importantly, the model predicted new regulatory interactions (such as the unexpected inhibition of *wnt* by *notum* recently validated in vivo) (Kakugawa et al. 2015), the existence of novel regulatory genes (unknown genes labeled *a* and *b* in the model and characterized as the Frizzled family of receptors [pending validation] and *hnf4* [manuscript in preparation], respectively), and the specific phenotypes produced after novel perturbations (such as the ability of *hnf4* to rescue abnormal phenotypes [manuscript in preparation]). Indeed, this methodology can readily assist in the definition of comprehensive models directly from the ever‐growing experimental datasets obtained at the bench, and hence accelerate our complete understanding of planarian regeneration. While the specific details of the model will continue to be tested and refined in planaria, this general scheme can be applied to many other models in regenerative biology (e.g., limb regeneration [Lobo et al. 2014a] and bioelectric induction of metastasis [Lobikin et al. 2015a]).

## Conclusions

The planarian regeneration field is at an extremely exciting place, poised to contribute to our understanding of physiological networks in pattern formation and evolution, as well as drive regenerative medicine advances. A few specific areas for future focus include (1) the continued development of comprehensive databases of planarian results, encompassing functional and physiological data, going beyond protein/gene datasets toward a bioinformatics of shape, and a standardization that will facilitate new machine learning approaches (Lobo et al. 2013a; Brandl et al. 2015); (2) novel monitoring and functional modification techniques, especially for physiological pathways. The extension of optogenetics, a powerful tool for probing neural and bioelectric controls of regeneration (Bernstein et al. 2012; Adams et al. 2013, 2014), may need to wait until misexpression technology becomes available in planaria. However, recent drug‐only approaches to light control of ion channel activity (Chambers et al. 2006; Tochitsky et al. 2014) and immobilization techniques (Dexter et al. 2014) may offer a way around the current impasse. (3) Much more work is needed to unify bioelectric and biochemical signaling. In particular, bi‐directional regulatory loops between specific chemical pathways, chromatin state, and spatial voltage distributions need to be characterized. Physiological networks also need to start being incorporated into the advanced modeling platforms, which heretofore largely focus on gene regulatory networks and biochemical gradients (Lobo & Levin 2015; Werner et al. 2015). (4) The molecular investigation of additional species of planaria (Sheiman et al. 2010) will facilitate studies of the evolutionary implications of bioelectric signaling. (5) Work on transplantation (Nodono & Matsumoto 2012), currently a technique only mastered in very few laboratories, will be essential to test questions of target morphology (Fig. 1), as well as to understand the relative contributions of neoblasts versus surrounding soma for specification of pattern. (6) A major question concerns the persistence of morphologies (such as two‐head forms). Aberrant forms are rarely re‐cut in studies, and it is unclear currently which of the many phenotypes exhibited in the literature are in fact permanent, or what mechanisms mediate the permanence. (7) The role of the CNS in regeneration is well known (Kumar & Brockes 2012), although the fact that it can be instructive for shape and not merely permissive (Mondia et al. 2011) is less often mentioned. In planaria, ventral nerve cord integrity synergizes with GJC to determine whether a head forms at a wound (Oviedo et al. 2010); this interaction is currently not understood but is probably a gateway to understanding the relationship between neural and non‐neural bioelectric signaling in pattern control. (8) Planaria are an emerging model for cancer (Oviedo & Beane 2009); interestingly, anterior regeneration has the ability to cure posterior tumors (Seilern‐Aspang & Kratochwil 1965), exhibiting another example of long‐range patterning influence. Given the recent advances in bioelectrics as a functional regulator of cancer (Lobikin et al. 2012; Chernet & Levin 2013a; Yang & Brackenbury 2013; Huang & Jan 2014), planaria may well be a very fruitful context in which to understand the physiological inputs into the tension between robust patterning morphostasis and the patterning disorganization of tumorigenesis. (9) Planarian behavioral capabilities extend far past sensory systems and even extend into learning and memory (McConnell 1965; Nicolas et al. 2008). Planaria demonstrate classical conditioning, instrumental learning, and can be preference trained (Thompson & McConnell 1955; Best & Rubenstein 1962; Wells 1967; Abbott & Wong 2008). Indeed, planaria are a unique model species in which memory and brain regeneration can be done in the same animal. This gives unprecedented opportunity to study the dynamics of memories during brain regeneration (McConnell et al. 1959; Shomrat & Levin 2013; Blackiston et al. 2015b).

One of the major areas for future development, in addition to specific techniques and datasets, is advances in conceptual integration of molecular data and algorithmic understanding of the regenerating body as a computational distributed system (Couzin 2007). Having seen the molecular conservation of information processing machinery (ion channels, GJs, and neurotransmitters) between brain function and planarian regeneration, it may be conjectured that some of the algorithms by which cell networks make decisions could also be conserved (Pezzulo & Levin 2015). We are currently testing this hypothesis by attempting to link realistic cell‐level electrophysiological simulation (Law & Levin 2015) to models of emergent patterning (Bessonov et al. 2015; Tosenberger et al. 2015) and dynamic systems descriptions of anatomical attractors (Friston et al. 2015), attempting a multi‐scale understanding of the planarian's remarkable shape homeostasis.

The planarian system is teaching us crucial lessons about how self‐repairing structures can be implemented via crosstalk between the genome and physical forces. Much of what we have seen in this model is highly conserved to bioelectric controls in vertebrate (Levin 2014a, 2014b) and even mammalian (Zhao et al. 2006; Lange et al. 2011) systems; these advances will suggest transformative roadmaps for biomedicine. Moreover, this invertebrate will teach us not only about this specific example of cell biology but more broadly about how communication among networks of agents implements adaptive pattern control (Levin 2012b). The impact of physiological studies in planaria, by revealing new ways to achieve guided self‐assembly of complex self‐repairing shapes, will probably impact artificial life, robotics, unconventional computation platforms, and the design of hybrid artificial agents (Doursat 2006; Doursat et al. 2013; Doursat & Sanchez 2014).
